# Preparation of a Novel Zirconium-Loaded Flocculant for Efficient Removal of Phosphorus

**DOI:** 10.3390/ma19102049

**Published:** 2026-05-14

**Authors:** Xueqing Xi, Xiang Li, Sufang He, Jiacheng Li, Boxuan Li, Xiangqian Zheng

**Affiliations:** 1 Faculty of Materials Science and Engineering, Kunming University of Science and Technology, Kunming 650093, China; 19862129557@163.com (X.X.); yunlix99@163.com (X.L.); sufanghe@kust.edu.cn (S.H.); 2 The Innovation Team for Volatile Organic Compounds Pollutants Control and Resource Utilization of Yunnan Province, Kunming 650500, China; 19096018218@163.com; 3 The Higher Educational Key Laboratory for Odorous Volatile Organic Compounds Pollutants Control of Yunnan Province, Kunming 650500, China; 4 Faculty of Chemical Engineering, Kunming University of Science and Technology, Kunming 650500, China; 5 Institute for Inspection and Certification of Xishuangbanna Dai Autonomous Prefecture, Jinghong 666100, China

**Keywords:** phosphorus removal, coagulation, polysilicate-ferric-aluminum-zirconium, composite flocculant, excellent removal performance

## Abstract

Polysilicate-ferric-aluminum-zirconium (PSFAZ) was prepared using co-polymerization for the treatment of phosphorus wastewater. The preparation conditions of PSFAZ were optimized through a series of single-factor experiments, including Zr/Fe molar ratio, pH, sedimentation time, and dosage. The results demonstrated that PSFAZ exhibited an excellent phosphorus removal performance with 99.3% removal efficiency under the conditions of Zr/Fe ratio of 0.6/1, pH of 6, dosage of 25 mL/L and sedimentation time of 2 h. In real wastewater treatment, PSFAZ exhibited an exceptional phosphorus removal efficiency of 99.6%, accompanied by negligible metal leaching. The characterization results reveal that charge neutralization, ligand exchange, bridging effect and complexation reactions between metal ions and phosphorus play a dominant role in phosphorus removal. This study provides valuable insights into the practical application of novel inorganic composite flocculants for phosphorus wastewater treatment and reuse.

## 1. Introduction

Phosphorus is now widely used in various industries, including chemical [1], fertilizer [2], pharmaceutical [3] and other fields. However, during phosphorus utilization, numbers of phosphorus-related pollutants are released into natural bodies without adequate monitoring and treatment. In recent years, overdischarge of phosphorus wastewater has become a severe environmental issue. Excess phosphorus can significantly affect the aquatic environment, leading to eutrophication, algal blooms [4], oxygen depletion and dead zones [5,6]. According to the China’s national discharge standard of pollutants for municipal wastewater treatment plant, the upper limit for total phosphorus is set as 0.5 mg/L (class 1A) [7]. However, research indicates that total phosphorus concentration exceeding 0.039 mg/L in aquatic ecosystems can trigger the loss of sensitive species. This suggests that the current discharge standard is inadequate for protection of the ecological integrity [8]. Therefore, it is crucial to develop practical measures to effectively remove phosphorus in wastewater. In previous studies, biological treatment [9], adsorption [10], advanced oxidation [11], membrane filtration [12], and coagulation [13] were considered to be effective phosphorus removal technologies. Among these, coagulation has emerged as a leading method among these technologies due to its excellent coagulation performance [14] and cost-effectiveness [15]. Zong et al. [16] synthesized coral reef-like flocs formed by a continuous dosing coagulation (CDC) process to investigate their coagulation performance towards phosphate. When the CDC dosage was 2 mg Al/L (2 mg of aluminum salt was added to every 1 L of wastewater), the total phosphorus residual concentration was extremely low (0.035 mg/L). Zhang et al. [17] applied a chitosan-modified bentonite flocculant to the treatment of high-concentration organic wastewater. Under optimal conditions, the removal rate of total phosphorus reached 52.3% owing to its strong bridging effects. Until now, polysilicate composite flocculants have undergone rapid development and are considered as promising phosphorus removal agents. Compared to traditional coagulants, polysilicate composite flocculants exhibit lower health risks and higher removal efficiencies. Xu et al. [18] developed a polysilicate ferric flocculant (PSF) synthesized from steel pickling waste liquor and industrial sodium silicate for the remediation of high-turbidity tailings wastewater. The results showed that transmittance of the kaolin suspension reached 94.4% at a dosage of 0.8 g/L. Liu et al. [19] synthesized inorganic biopolymers by grafting sodium alginate onto polysilicate aluminum calcium (PSAC) via coordination bonding, and subsequently evaluated their performance in removing turbidity and color from aqueous solutions. The results demonstrated that the maximum turbidity and color removal efficiency reached 97.2% and 98.4% for the kaolin-humic acid suspensions, respectively. Despite their widespread application in wastewater treatment, these conventional ferric and aluminum-based coagulants exhibit inherent limitations that constrain their treatment efficiency. The coagulation performance of these flocculants is constrained by poor stability [20] and complex synthesis routes [21]. Moreover, the inappropriate flocculants can result in the production of substantial hazardous sludge, which subsequently requires additional disposal procedures [22]. By contrast, the introduction of zirconium offers a viable strategy to overcome this inherent constraint. Li et al. [23] reported that polymeric zirconium sulfate achieves turbidity removal exceeding 95% and total phosphorus removal exceeding 90%. The superior removal efficiency was ascribed to the coagulant’s wide pH tolerance, which derives from the unique hydrolysis behavior of zirconium species. Notably, zirconium-based compounds are widely recognized for their high affinity for phosphate, non-toxicity, and stability. These characteristics render them suitable candidates for wastewater treatment applications [24]. Based on that, it is necessary to integrate these approaches and develop a compelling strategy for enhanced phosphorus removal. In this work, a zirconium-loaded flocculant was prepared via co-polymerization, and its phosphorus removal performance was investigated. Polysilicates doped with ferric (PSF), ferric-aluminum (PSFA) and ferric-aluminum-zirconium (PSFAZ) were synthesized to remove phosphate from simulated phosphorus wastewater. Furthermore, its coagulation performance was optimized by evaluating the Zr/Fe molar ratio, dosage, pH, and sedimentation time. Overall, this novel inorganic polymer metal salt demonstrates significant potential in phosphorus wastewater treatment.

## 2. Materials and Methods

### 2.1. Preparation of Chemicals and Reagents

All the chemicals and reagents involved in this study are shown in Supplementary Materials Section S1.

### 2.2. Preparation of Flocculants

All the flocculants were synthesized at room temperature. Comprehensive compositions of each flocculant are shown in Table 1. The flocculants were prepared as follows. First, a hydrochloric acid solution (1 + 1) was prepared by mixing 36 wt% hydrochloric acid and ultrapure water in a 1:1 volume ratio (*v*/*v*). Hydrochloric acid (1 + 1) was slowly added dropwise to 200 mL of sodium silicate solution with 1.5% (*w*/*v*) silica content, under a very slow stirring rate. According to our previous study [25], to ensure a high degree of polymerization of polysilicic acid (PSi), the optimal silica content and synthesis pH were 1.5% and 2, respectively. Accordingly, the pH of the mixture was constantly monitored until it reached 2. Afterward, the agitation was continued for another 2 h at 60 rpm, resulting in the formation of the polysilicic acid (PSi) solution. This step enables sodium silicate to polymerize and polysilicic acid (PSi) is prepared. The obtained solution was then moderately stirred to improve its degree of polymerization. After the polymerization process, 0.05 mol (6.7 g) of zirconium chloride (ZrCl_4_), 0.05 mol (13.6 g) of ferric chloride hexahydrate (FeCl_3_⋅6H_2_O) and 0.05 mol (18.8 g) of aluminum nitrate nonahydrate (Al(NO_3_)_3_⋅9H_2_O) were added to the PSi solution to obtain polysilicate-ferric-aluminum-zirconium (PSFAZ). Polysilicate-ferric (PSF) was obtained by adding 0.05 mol (13.6 g) ferric chloride hexahydrate (FeCl_3_⋅6H_2_O) into polysilicic acid (PSi) solution. Polysilicate-ferric-aluminum (PSFA) was prepared by adding 0.05 mol (13.6 g) ferric chloride hexahydrate (FeCl_3_⋅6H_2_O) and 0.05 mol (18.8 g) of aluminum nitrate nonahydrate (Al(NO_3_)_3_⋅9H_2_O) into polysilicic acid (PSi) solution. In order to guarantee an excellent metal loading performance, three flocculants were subjected to an additional 2 h of continuous sedimentation without stirring. After 2 h of sedimentation, the liquid-state flocculants were successfully prepared.

### 2.3. Phosphorus Coagulation Experiments

In order to investigate the effect of the Zr/Fe molar ratio, pH, dosage, and sedimentation time on phosphorus removal, a series of single-factor coagulation experiments were conducted. An optimal Zr/Fe molar ratio can ensure an excellent phosphorus removal performance. Excessive amount of zirconium content demonstrated low cost-effectiveness, inadequate zirconium content led to poor phosphorus removal performance. In this study, the Zr/Fe molar ratio was adjusted to 0.2/1, 0.4/1, 0.6/1, 0.8/1 and 1/1 by adding varied amounts of zirconium chloride into the polysilicic acid (PSi) solution. pH plays a crucial role in phosphorus removal owing to its significant influence on the flocculant. An extremely high pH environment can cause competitive coagulation between OH^−^ ions and phosphate, weakening the process of phosphorus removal. In this study, the pH of the solution was set to 2, 3, 4, 5, 6, 7, 8, 9, 10 and 11. Effect of dosage is a key factor in phosphorus removal. Insufficient dosage was not conductive to the removal of phosphorus since there was limited metal ions to bind with phosphate. On the contrary, excessive dosage may lead to an “ion binding” effect which eventually impairs the coagulation ability of the flocculant [26]. Accordingly, the dosages were set to 10, 15, 20, 25, 30, 35, 40 mL/L by adding varying amounts of PSFAZ to the simulated wastewater solution. An appropriate sedimentation time ensures a high degree of polymerization of the flocculant which can improve its phosphorus removal performance [27]. In order to investigate the removal efficiency of PSFAZ under varying sedimentation time conditions, the sedimentation time was adjusted to values of 0.5, 1, 2, 4, 8 and 12 h. A comprehensive set of experimental conditions were shown in Table 2. All coagulation experiments were conducted in plexiglass beakers using a magnetic stirrer, following a three-step procedure: rapid mixing, slow stirring and stationary sedimentation. During the rapid mixing process, the suspension was stirred at 200 rpm for 2 min, with a quantitative coagulant dose and the pH was adjusted using 1 mol/L hydrochloric acid (HCl) and 1 mol/L sodium hydroxide (NaOH). This was followed by slow stirring at 60 rpm for 5 min. After the slow stirring period, the mixture was allowed to settle for 30 min. Following 30 min of the stationary sedimentation process, supernatant samples were collected from a depth of 2–3 cm below the water surface using a syringe and subsequently filtered through a 0.45-μm membrane filter prior to the total phosphorus concentration analysis. The removal rate (*R*%) for total phosphorus was obtained using Equation (1) [28].


(1) $$R\%=\frac{C_{0}-C_{e}}{C_{0}}\times 100\%$$

In the equation above, C_0_ stands for initial concentration of total phosphorus, C_e_ stands for the concentration of total phosphorus at equilibrium and R% stands for phosphorus removal rate.

### 2.4. Characterizations of Flocculants

The concentrations of total phosphorus and metal ion leakage were determined using ICP-OES (Agilent 5800, Agilent, Melbourne, Australia). The TSS of real wastewater sample was measured using gravimetric method (GB/T 11901-1989) [29]. The turbidity of real wastewater sample was determined by a turbidimeter (WGZ-200BS, Hangzhou, China). COD of real wastewater samples was measured by digestion-spectrophotometric method (HJ/T 399-2007) [30]. Zeta potential of the flocculant and real wastewater were determined by Zetasizer Nano-ZS90 (Malvern, UK), samples were dispersed in deionized water at 25 °C, injected into a folded capillary cell, and subjected to electrophoretic light scattering, where phase analysis light scattering (PALS) determined electrophoretic mobility, then converted to zeta potential via Smoluchowski model. Fourier transform infrared spectroscopy (FTIR) was conducted to analyze the solid samples of the flocculants by using a Bruker Invenio-s spectrometer (Bruker, Billerica, MA, USA) in the range of 4000–400 cm^−1^. Three flocculants’ crystallinity before coagulation and PSFAZ’s crystallinity under different n(Zr)/n(Fe) molecular ratio (0.2/1–1/1) were determined by X-ray diffraction (XRD). It was performed using X’pert 3 powder (Malvern-Panalytical, Malvern, UK) for the determination of crystallinity with Cu Kα radiation in the 2θ range of 20–80° at a scan rate of 4° min^−1^. The microstructures of the flocculants and their elemental compositions were examined using a Nova Nanosem 450 Scanning Electron Microscope (SEM) (Thermo Fisher, Waltham, MA, USA). The X-ray photoelectron spectroscopy spectra were obtained using X-ray photoelectron spectroscopy (ULVAC PHI, Shanghai, China) with Al Kα radiation (hv = 1486.6 eV). The solution pH was measured using a pH meter (PHS-3C, Shanghai, China). All the data analysis was processed using the Origin 2021 software (Northampton, MA, USA).

### 2.5. Statistical Analysis

One-way analysis of variance (ANOVA) followed by Fisher’s least significant difference (LSD) post hoc test (α = 0.05) was employed to evaluate significant differences in the removal of phosphorus under varied conditions (Zr/Fe molar ratio, pH, dosage, sedimentation time, coexisting anions, zeta potential and metal leakage). Optimization experiments were performed in triplicate (*n* = 3), and results are presented as mean ± standard deviation (SD). All statistical analysis were conducted using Origin 2021 software (Northampton, MA, USA).

## 3. Results and Discussion

### 3.1. Morphology and Structure

#### 3.1.1. SEM-EDS

**Figure 1:**
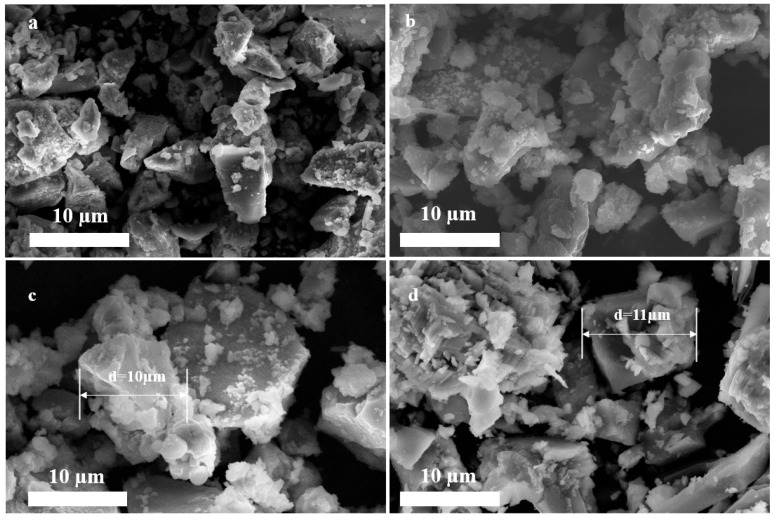
SEM images of (**a**) PSF; (**b**) PSFA; (**c**) PSFAZ; (**d**) PSFAZ after coagulation.

SEM images of the PSF, PSFA, and PSFAZ are shown in Figure 1. Figure 1a (scale bar = 10 µm) shows that the PSF had a discrete structure with relatively smooth surface, demonstrating the low bridging effect of PSF. Compared with PSF, there were more scattered particles observed on the surface of PSFA, as shown in Figure 1b (scale bar = 10 µm). Furthermore, the compressed structure of PSFA shows a trend of cluster. Figure 1c (scale bar = 10 µm) shows a brick-like shape and a porous multi-branch system. The incorporation of three metal salts resulted in a roughened surface morphology and an expanded branch structure in PSFAZ, whereas such features were absent in PSF and PSFA. Its high specific surface area and dense structure enable PSFAZ to adsorb and bond phosphate ions within the wastewater. Moreover, the introduction of polysilicic acid increased the molecular weight of the flocculant, which is conducive to the bridging effect and sweep flocculation. If the synthesized flocculant is predominantly composed of macromolecular substances, it generally exhibits enhanced coagulation efficiency toward the target pollutants. These macromolecules can amplify the bridging effect, thereby facilitating the formation of flocs. As a result, the flocs became easily settleable and were conducive to further sedimentation [23]. SEM image of the PSFAZ after coagulation is shown in Figure 1d (scale bar = 10 µm). The porous skeleton structure of PSFAZ was covered by irregularly shaped particles, indicating the occurrence of adsorption, charge neutralization, and bridging effect. As a whole, the multi-branch system and rough surface of the PSFAZ is conductive to floc formation and its subsequent sedimentation process. Figure S1 illustrates the distribution of the PSFAZ elements before and after coagulation. The results showed that all metal elements were successfully doped and uniformly dispersed. Energy dispersive X-ray spectroscopy (EDS) analysis was further employed to determine elemental composition before and after coagulation. The peak of phosphorus was detected in the EDS mapping, confirming the successful capture of phosphorus onto the flocculant during the coagulation process.

#### 3.1.2. XRD Analysis

**Figure 2:**
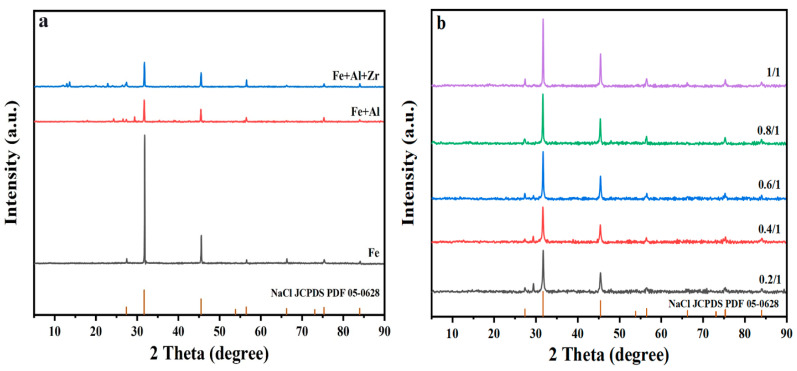
(**a**) XRD pattern of PSF, PSFA and PSFAZ; (**b**) XRD pattern of PSFAZ with different n(Zr)/n(Fe) molecular ratio after coagulation.

The XRD patterns of PSF, PSFA and PSFAZ are presented in Figure 2a. The results reveal that the XRD patterns of three materials performed similar trends. From these patterns, the diffractive bands of Fe, Al or Zr compounds cannot be observed, which may be attributed to the amorphous nature of the material. The diffraction bands observed at approximately 32° and 46° were attributed to NaCl, which formed as a by-product during sample dehydration [31]. The XRD spectra of PSFAZ with different n(Zr)/n(Fe) molar ratios are shown in Figure 2b. There is no crystal phase difference observed compared with the XRD spectra before coagulation. Moreover, all the XRD spectra under different n(Zr)/n(Fe) molar ratios are similar, indicating the good structural stability of PSFAZ.

### 3.2. Evaluation of Coagulation Performance

#### 3.2.1. Comparison of the Removal Efficiencies of Various Flocculants

**Figure 3:**
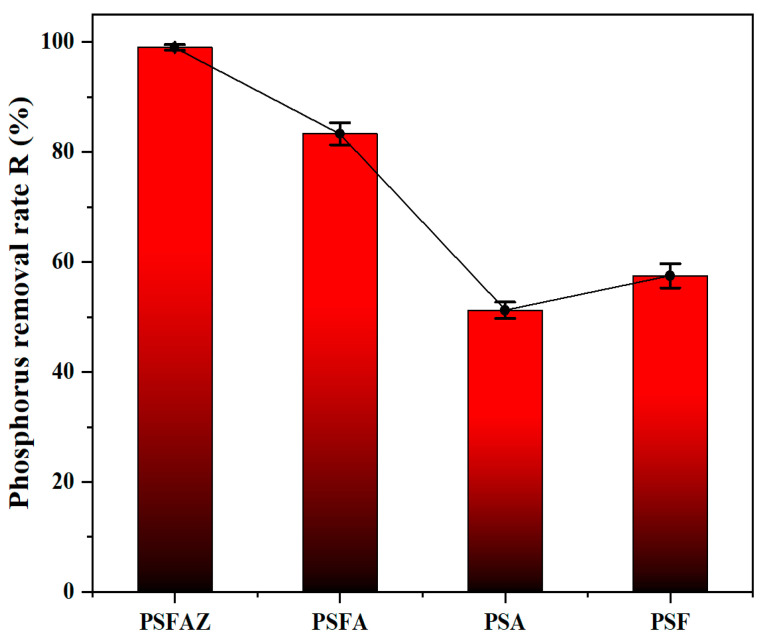
Comparison of the removal efficiencies of four flocculants (Zr/Fe molar ratio = 0.6/1, pH = 6, dosage = 25 mL/L, Sedimentation time = 2 h, Sample size *n* = 3).

Figure 3 presents a comparative analysis of total phosphorus removal performance of PSF, PSA, PSFA and PSFAZ in simulated phosphorus wastewater. The same dose of flocculant (25 mL/L) was added to the simulated phosphorus wastewater under room temperature (25 °C). Each experiment was duplicated three times and the average removal efficiency result was obtained. The removal efficiency of PSA and PSF were 51.2% and 57.5%, respectively. PSFA exhibited a moderate removal efficiency of 83.3%, which was significantly higher than that of PSA or PSF alone but still inferior to that of PSFAZ. The main reason could be its low affinity for phosphate. Unlike Zr^4+^, which forms stable inner-sphere complexes, Fe^3+^ and Al^3+^ tend to form outer-sphere complexes or simple precipitates that are less stable and more easily dissociated [32]. The introduction of zirconium remarkably improved the phosphorus removal efficiency of PSFAZ compared with PSF, PSA, and PSFA, with a phosphorus removal efficiency of 99.3%. Owing to the enhanced surface charge density of Zr-based flocculant, PSFAZ could readily form stable complexes with phosphate ions, resulting in exceptional phosphorus removal efficiency even at trace concentrations [33]. The observed high selectivity towards phosphorus is attributed to the specific interaction between the positively charged sites on zirconium and the long pairs of phosphate ions, which facilitates the formation of inner-sphere complexes while excluding other co-existing anions [34]. The addition of PSFAZ resulted in the rapid aggregation of suspended solids. Visual evidence of the floc formation was shown in the Supplementary Materials (Figure S2).

#### 3.2.2. Effect of Zr/Fe Molar Ratio

**Figure 4:**
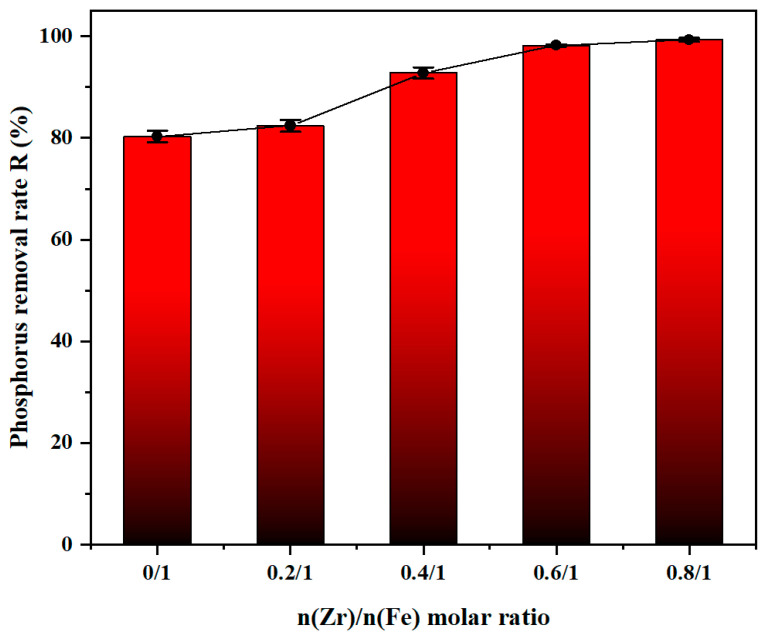
Effect of Zr/Fe molar ratio towards the removal of phosphorus (pH = 6, dosage = 25 mL/L, sedimentation time = 2 h, sample size *n* = 3).

The Zr content within the flocculant plays a critical role in determining the removal efficiency of phosphorus. Low zirconium content within the flocculant will lead to a low degree of polymerization, which subsequently demolishes the binding effect between zirconium and phosphates within the wastewater. Figure 4 illustrated that the coagulation efficiency was highly dependent on the Zr/Fe molar ratio. In the absence of zirconium, the removal efficiency of phosphorus is only 80.32% by PSFA. As the Zr/Fe molar ratio increases from 0.2/1 to 0.6/1, the removal efficiency continuously increases from 82.43% to 98.2%. The removal efficiency exceeds 99% when the Zr/Fe molar ratio reached 0.8/1 and remained steady as the Zr/Fe molar ratio increased to 1/1. The introduction of zirconium enhances charge neutralization and inner-sphere complexation towards phosphorus, thereby effectively capturing phosphate [35]. While higher Zr/Fe ratios (>0.6/1) lead to only marginal improvements in removal efficiency, the cost of zirconium salts is approximately 15 times that of iron salts and 13 times that of aluminum salts. The Zr/Fe ratio of 0.6/1 reduces material cost by about 40% relative to the 0.8/1 ratio, while still achieving >98% removal efficiency. To guarantee an optimal coagulation performance and good cost-effectiveness, the Zr/Fe ratio of 0.6/1 was selected in the subsequent experiments.

#### 3.2.3. Effect of pH

**Figure 5:**
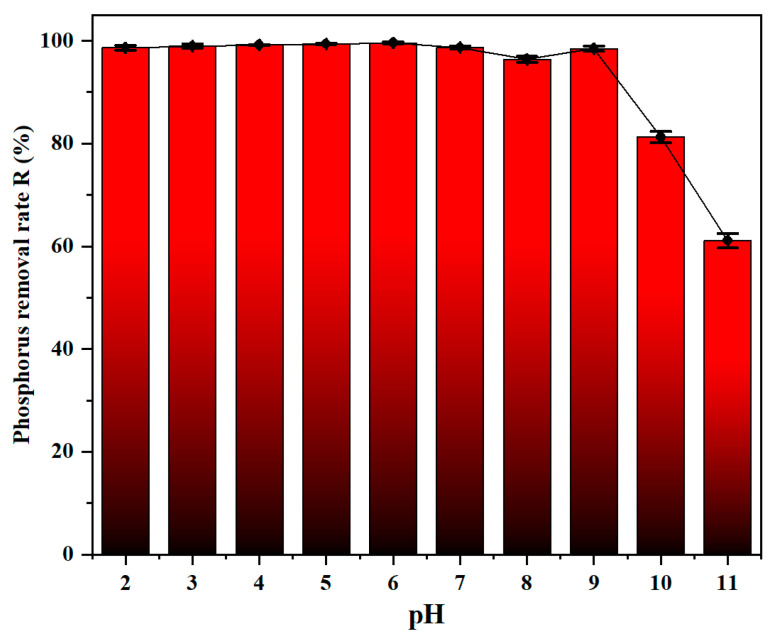
Effect of pH towards the removal of phosphorus (Zr/Fe molar ratio = 0.6/1, dosage = 25 mL/L, sedimentation time = 2 h, sample size *n* = 3).

The efficiency of phosphorus removal is well-known to be strongly influenced by the solution pH [36]. In this work, the effect of pH on phosphorus removal efficiency is evaluated from 2 to 11, as illustrated in Figure 5. The results have shown that the composite flocculant exhibited its worst coagulation performance at pH = 11. In contrast, it maintains a good coagulation performance within the pH range of 2 to 6 and the phosphorus removal rate remained between 98.6% and 99.5%. Under acidic conditions, charge neutralization is the dominant mechanism. The flocculant undergoes hydrolysis to form polynuclear hydroxyl complex ions, which enables these ions to absorb the negatively charged particles, thereby enhancing the ability of charge neutralization [37]. As pH increases from 6 to 8, the overall removal efficiency fluctuated and decreased from 99.5% to 93.1%. Within this pH range, the positive charge of Zr ions gradually diminishes, thereby weakening charge neutralization. The dominant mechanism shifts to a synergistic effect of bridging and inner-sphere complexation. According to XPS analysis, the presence of the Zr-O-P complex and the decrease in relative content of M-OH components confirms the strong effect of inner-sphere complexation. Meanwhile, PSi polymerizes into a stable three-dimensional network with a long-chain branched morphology, enabling it to simultaneously adsorb multiple colloidal particles and effectively connect them into large, dense flocs. As pH further increases from 8 to 11, the removal efficiency dropped from 93.1% to 61.1%. Under alkaline conditions, the negative surface charge induced electrostatic repulsion, which suppressed charge neutralization. Moreover, PFSAZ reacts with excess OH^−^ ions, accelerating the hydrolysis of metal ions. These ions compete with phosphate for metal binding sites and promote the formation of metal hydroxide precipitates rather than metal–phosphorus complexes [38]. Additionally, the diminished coagulation performance involves the partial destruction of the polysilicic acid skeleton through Si-O-Si bond rupture, which compromises its bridging ability and thereby reduces phosphorus removal efficiency [39]. Accordingly, a pH of 6 was employed as the optimal condition for subsequent coagulation experiments.

#### 3.2.4. Effect of Dosage

**Figure 6:**
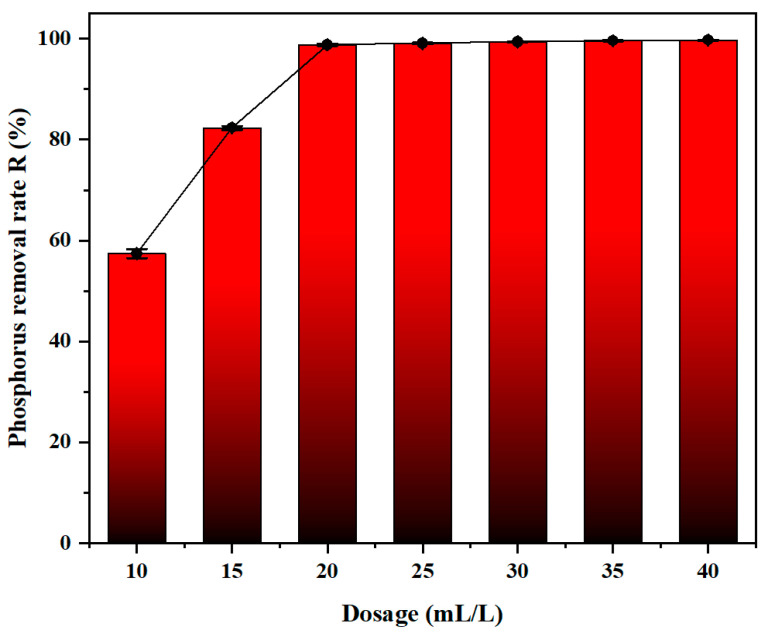
Effect of dosage towards the removal of phosphorus (Zr/Fe molar ratio = 0.6/1, pH = 6, sedimentation time = 2 h, sample size *n* = 3).

As shown in Figure 6, dosage is a key factor in affecting flocculant’s coagulation performance. When the dosage is 10 mL, the phosphorus removal efficiency is 57.4%. As the dosage increased from 15 mL to 25 mL, the removal efficiency increased remarkably, eventually exceeding over 99%. Finally, when the dosage increased from 30 mL to 40 mL, the overall removal efficiency remained steady. Once the flocculant was released, it undergoes rapid hydrolysis and interacts with anionic ions through electro-static attraction. However, an excess of flocculant can coat the surfaces of metal particles, causing a charge reversal effect, thereby weakening charge neutralization and reducing the removal efficiency of total phosphorus [26,40]. Based on that, the optimal dose was determined to be 25 mL/L.

#### 3.2.5. Effect of Sedimentation Time

Since the degree of polymerization is considerably affected by sedimentation time, the influence of this parameter on phosphorus removal efficiency was examined over a range of 0.5 to 12 h (Figure S3). As sedimentation time increased from 0.5 to 2 h, the removal efficiency correspondingly rose from 76.6% to 99.8%. The overall removal efficiency remained constant across the time range of 4 to 12 h. Consequently, a sedimentation time of 2 h was identified as optimal for phosphorus removal. An appropriate sedimentation time would allow more active components to be dispersed into the polysilicic acid (PSi) network structure, subsequently enhancing its phosphorus removal performance through inner-complexation and charge neutralization [41].

### 3.3. Real Wastewater Treatment

#### 3.3.1. Effect of Coexisting Anions

**Figure 7:**
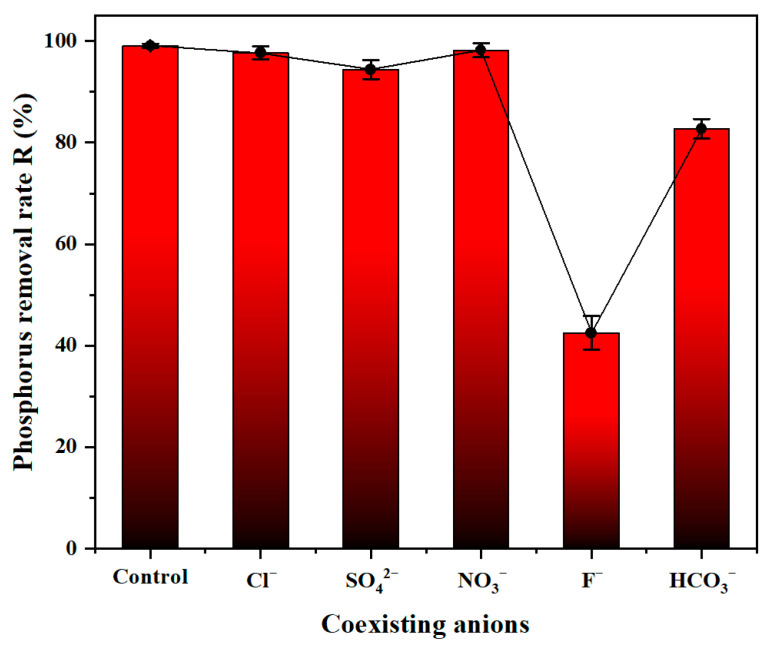
Effect of coexisting anions towards the removal of phosphorus (5 anions’ concentration = 50 mg/L, initial TP concentration = 200 mg/L, Zr/Fe molar ratio = 0.6/1, pH = 6, dosage = 25 mL/L, sedimentation time = 2 h, sample size *n* = 3).

In real wastewater treatment, various coexisting anions can significantly affect phosphorus removal performance. Therefore, investigating their influence is necessary to facilitate the practical application of PSFAZ. In our study, the coagulation performance of PSFAZ was evaluated in the presence of Cl^−^, SO_4_^2−^, NO^3−^, F^−^ and HCO^3−^, each at a fixed concentration of 50 mg/L. The influence of the five anions towards phosphorus removal was shown in Figure 7. In general, the inhibitory effect on phosphorus removal by PSFAZ was ranked as NO^3−^ < Cl^−^ < SO_4_^2−^ < HCO^3−^ < F^−^. There was no obvious effect on the phosphorus removal in the presence of Cl^−^, NO^3−^ and SO_4_^2−^, with removal efficiencies of PSFAZ remaining at 97.7%, 98.2% and 94.4%, respectively. This can be attributed to their poor ligands for inner-sphere complexation compared to phosphate species, which form stable M-O-P bonds via ligand exchange. The inhibitory effect of HCO^3−^ was moderate compared with Cl^−^, NO^3−^ and SO_4_^2−^, with a decrease in removal efficiency of 16.4%. HCO^3−^ may increase the solution pH (i.e., HCO^3−^ + H_2_O → H_2_CO_3_ + OH^−^), resulting in weakened electrostatic attraction to anionic phosphate species [42]. Fluoride ions had the most detrimental impact on coagulation performance, with a decrease in removal efficiency of 55.6%. Fluoride ions preferentially bind to the metal-hydroxyl sites (M–OH) on PSFAZ, forming soluble [MF_x_]^n−^ species or surface complexes that block active sites and prevent the ligand exchange required for phosphate uptake, which results in significant reduction of phosphorus removal efficiency [43]. Overall, despite the unsatisfactory removal efficiency in the presence of F^−^, PSFAZ is still considered a possible candidate for real wastewater treatment.

#### 3.3.2. Coagulation Performance in Rubber Wastewater

**Figure 8:**
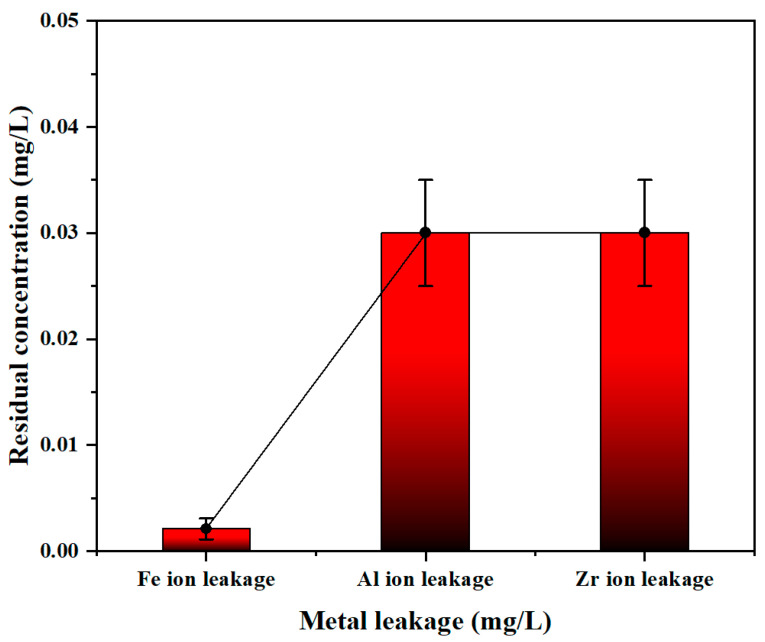
Metal leakage in real rubber wastewater (Zr/Fe molar ratio = 0.6/1, pH = 6, dosage = 25 mL/L, sedimentation time = 2 h, sample size *n* = 3).

Wastewater samples were obtained from a local raw rubber manufacturing plant in Xishuangbanna, its characteristics are summarized in Table 3 (residual TP concentration was also included in Table 3). Based on the preliminary investigation, the content of organic matter and other co-existing anions within the wastewater is negligible, total phosphorus is identified as the target pollutant. PSFAZ was selected as the flocculant to evaluate its removal performance towards phosphorus in rubber wastewater. The experiments were conducted in optimal experimental conditions (Zr/Fe molar ratio = 0.6/1, pH = 6, dosage = 25 mL/L, sedimentation time = 2 h). Meanwhile, the residual metal ion concentrations were monitored throughout the treatment process, with results presented in Figure 4. The result shows that the phosphorus removal efficiency of PSFAZ has achieved 99.6% (Table 3). Figure 8 presents the residual concentrations of Fe, Al, and Zr ions after coagulation, which were measured as 0.002, 0.03, and 0.03 mg/L, respectively. These values indicate satisfactory stability of the PSFAZ coagulant. The observed extremely low metal leaching is primarily attributed to the crosslinking property of polysilicic acid (PSi) incorporated into the PSFAZ matrix. This crosslinked network physically entraps metal ions and chemically coordinates them, thereby minimizing their release into the aqueous phase. Furthermore, PSFAZ demonstrates considerable potential for phosphorus removal compared with other reported materials, as summarized in Table S1. Unlike polymeric Zr salts that rely solely on zirconium, PSFAZ integrates Fe, Al, Si, and Zr into a single hybrid structure. This synergy enhances charge neutralization and bridging effect, achieving superior phosphorus removal at high initial phosphorus concentrations. Compared with conventional Zr-modified polysilicates, PSFAZ covalently incorporates Zr into the Si–O–Fe/Al network during co-polymerization, improving stability under potential practical conditions. In addition, compared with biopolymer-based materials that are prone to metal leaching under acidic or alkaline conditions, PSFAZ maintains excellent chemical stability and high coagulation efficiency across a wide pH range. Biopolymer-based materials are prone to Zr leaching under acidic or alkaline conditions, whereas PSFAZ exhibits excellent chemical stability and maintains high coagulation efficiency across a wide pH range. Based on the synthesis route described in Section 2.2 and the use of industrial-grade chemicals (with market prices for each element within PSFAZ), the production cost for PSFAZ was $143 per 1 ton. The unit cost in raw rubber wastewater treatment is approximately $0.07 per 1 ton. A comparison with other commercial coagulants was presented in Table S2. The results demonstrate the favorable cost-effectiveness of PSFAZ for future practical applications. The phosphorus-containing sludge generated during the coagulation process can be directly treated as phosphate fertilizer in agricultural application, the primary limitation of this strategy lies in the potential failure of metal content within sludge to comply with regulatory standards for heavy metals. Alternatively, acid leaching [44] and wet chemical recovery [45] are considered as an efficient strategy in phosphorus recovery from sludge. Overall, PSFAZ exhibits excellent phosphorus removal performance and stability in rubber wastewater, highlighting its considerable potential in future practical applications.

### 3.4. Flocculation Mechanism

#### 3.4.1. Zeta Potential Analysis

**Figure 9:**
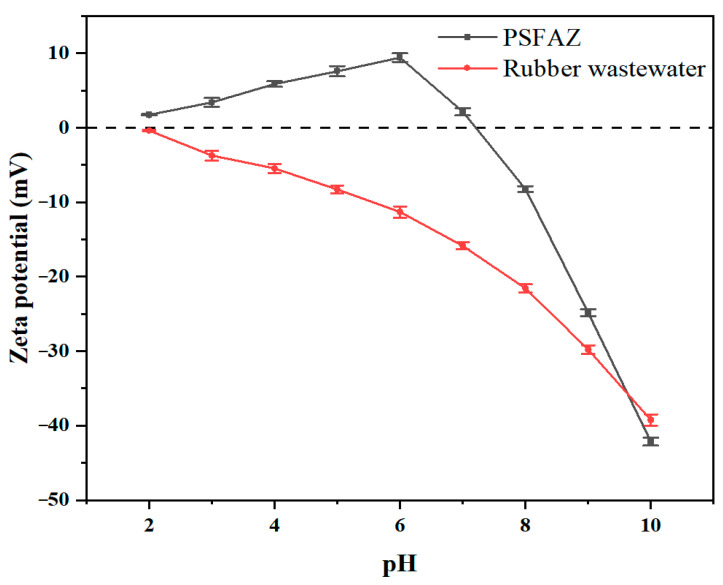
Effect of pH on zeta potential of PSFAZ flocculant and rubber wastewater.

Zeta potential serves as a critical parameter for elucidating the coagulation mechanism [46]. The zeta potential of PSFAZ and rubber wastewater was measured in the pH range of 2 to 10, with the results presented in Figure 9. Throughout the examined pH range, the zeta potential of rubber wastewater remained negative. In contrast, the zeta potential of PSFAZ increased progressively as the pH range increased from 2 to 7, reaching the highest zeta potential of 9.42 mV at pH = 6. In acidic conditions, Fe^3+^, Al^3+^ and Zr^4+^ ions can hydrolyze and produce positively charged particles, which promote the adsorption of negatively charged phosphate through electrostatic attraction [47]. Accordingly, electrostatic attraction is the main contributor to phosphorus removal in the pH range of 2 to 7. The isoelectric point of PSFAZ was observed between pH = 7 and 8. When the pH exceeds 8, PSFAZ becomes negatively charged, leading to an electrostatic repulsion that inhibits the interaction with phosphate species in wastewater. The phosphorus removal at pH > 8 was mainly accomplished by bridging effect and ligand exchange [48]. The specific removal mechanism was further investigated by FTIR and XPS analysis.

#### 3.4.2. FTIR Analysis

**Figure 10:**
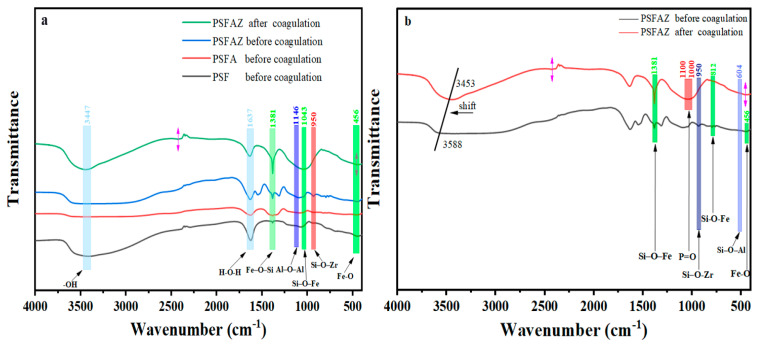
(**a**) FT-IR spectra of three flocculants before coagulation; (**b**) FT-IR spectra of PSFAZ flocculants before and after coagulation.

The FTIR spectra of three synthesized flocculants are presented in Figure 10a. A strong and broad absorption band observed at 3447 cm^−1^ corresponds to the stretching vibration of the -OH group [49], which indicates a large amount of -OH groups in the flocculant. The absorption band around 1637 cm^−1^ is attributed to the bending vibrations of absorbed, coordinated and crystal water molecules [29]. Meanwhile, the band at 1043 cm^−1^ may be associated with the overlapping vibrations of Si-O-Si and possibly Si-O-M (M = Fe, Al) [50]. The band at 1146 cm^−1^ is attributed to the vibration of the Al-O-Al group. The band at 456 cm^−1^ is attributed to the stretching vibration of the Fe-O group [51]. Notably, compared with PSF and PSFA, a new absorption band at 950 cm^−1^ was observed in PSFAZ, which is attributed to the bending vibration of the Si-O-Zr [52]. The emergence of this new band indicates that Zr has participated in phosphorus removal through complexation, thereby enhancing the phosphorus removal performance [53]. Figure 10b presents the FTIR spectra of PSFAZ before and after coagulation. The absorption band located at 1381 cm^−1^ and 604 cm^−1^ belongs to the bending vibrations of Si-O-Fe and Si-O-Al, respectively. The -OH stretching band shifted from 3447 cm^−1^ to a higher frequency of 3588 cm^−1^ during the coagulation process, indicating the interaction with phosphate. Meanwhile, the appearance of a P-O stretching band at 1000–1100 cm^−1^ has provided direct evidence of phosphorus uptake on PSFAZ. This interaction was further accompanied by the disappearance of bands at 950 cm^−1^, 812 cm^−1^ and 456 cm^−1^, indicating that both Zr and Fe have participated as active sites into phosphorus removal through complexation and charge neutralization.

#### 3.4.3. XPS Analysis

**Figure 11:**
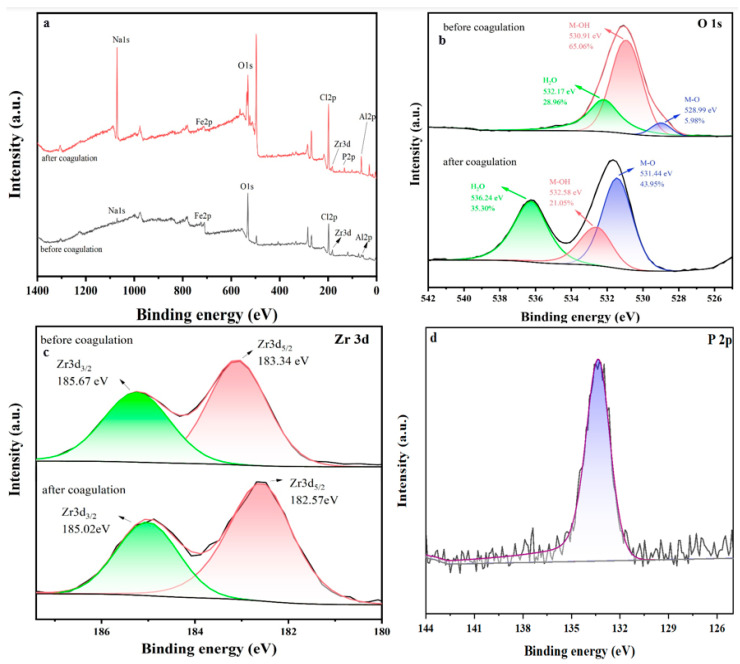
(**a**) Full XPS spectra of PSFAZ before and after coagulation; (**b**) O1s spectra of PSFAZ before and after coagulation; (**c**) Zr 3d spectra of PSFAZ before and after coagulation; (**d**) P 2p spectra of PSFAZ after coagulation.

XPS spectrum analysis is employed to characterize the surface chemical composition of the synthesized flocculant, as presented in Figure 11. The full XPS spectrum in Figure 11a confirms the existence of Zr, Fe, Al, and Fe, which is consistent with the FT-IR results. In Figure 11d, the emergence of P 2p located at 133.7 eV provides direct evidence of the successful phosphorus coagulation process [54]. High-resolution XPS spectra of O 1s before and after coagulation were deconvoluted into three distinct components, as presented in Figure 11b: three overlapping peaks corresponding to M-O (M = Fe, Al, Zr, 528.99 eV), M-OH (530.91 eV), and H_2_O (536.24 eV), respectively [55]. Upon phosphorus uptake, a significant reduction in the relative content of M-OH was observed, from 64.26% to 21.05%. This reduction confirms that ligand exchange is involved in phosphorus removal [56]. Additionally, the binding energy of M-O spectra shifted to a higher value from 528.99 eV to 531.44 eV, which is possibly associated with the formation of M-O-P inner-sphere complex through complexation reactions [57]. Furthermore, as shown in Figure 11c, after coagulation with phosphorus, the Zr 3d_3/2_ and Zr 3d_5/2_ spectra shifted to higher binding energies, indicating the formation of Zr-O-P precipitates. This phenomenon suggests that precipitation also contributes to phosphorus removal [58,59].

**Figure 12:**
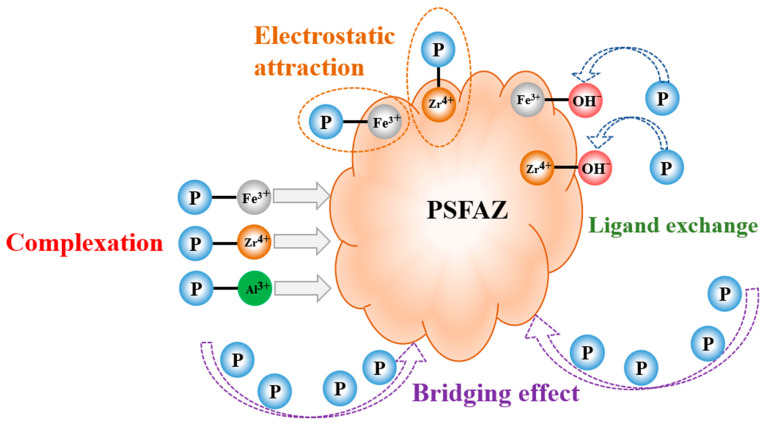
Schematic mechanism of phosphorus coagulation on PSFAZ.

A conceptual diagram representing the coagulation mechanism is depicted in Figure 12. After adding the positively charged composite flocculant PSFAZ to the simulated phosphorus wastewater, it interacts with colloidal particles through charge neutralization, thereby promoting the formation of flocs. PSFAZ spontaneously forms cross-linked structures, which not only enhances its bridging capacity but also promotes the formation of large flocs. As a whole, the involvement of Zr^4+^, Fe^3+^, and Al^3+^ cations towards phosphorus removal is confirmed by FTIR and XPS analyses, demonstrating the concurrent occurrence of complexation, ligand exchange, and co-precipitation mechanisms during the coagulation process.

## 4. Conclusions

In this study, a novel polysilicate-ferric-aluminum-zirconium (PSFAZ) flocculant was successfully prepared using co-polymerization for the effective removal of phosphorus from wastewater. Compared with PSF and PSFA, PSFAZ exhibited a superior coagulation performance with 99.3% removal efficiency of phosphorus. The optimum coagulation conditions of PSFAZ for phosphorus removal were as follows: dosage of 25 mL/L, sedimentation time of 2 h, pH of 6, and Zr/Fe molar ratio of 0.6/1. In addition, PSFAZ showed a good phosphorus coagulation efficiency in the presence of several coexisting anions (Cl^−^, SO_4_^2−^, NO^3−^ and HCO^3−^). In rubber wastewater treatment, PSFAZ has demonstrated outstanding phosphorus removal performance, achieving a removal efficiency of 99.6%. In addition, the metal leakage was effectively controlled under this situation, indicating the good stability of the flocculant. The underlying removal mechanisms were further explored by Zeta potential, FTIR and XPS analysis. Zeta potential test proved that electrostatic attraction plays a vital role in phosphorus removal under the pH range of 2 to 7. Additionally, ligand exchange, bridging effect and complexation also contributed to the phosphorus removal as evidenced by FTIR and XPS analysis. Despite its promising coagulation performance, the application of PSFAZ flocculant is hindered by several limitations. First, the inherent instability of polysilicic acid renders the composite prone to gelation during storage. Second, since this study only focuses on rubber wastewater with a low organic load, its applicability to other typical industrial wastewaters remains to be investigated. Therefore, future research should focus on improving the storage stability of PSFAZ and exploring its potential use in other industrial effluents. This work not only describes an innovative strategy for the design of inorganic composite flocculants, but also provides valuable insights for phosphorus wastewater treatment in sustainable water reuse.

## Figures and Tables

**Table 1:** Comprehensive compositions of each flocculant.

	Flocculants	PSF	PSA	PSFA	PSFAZ
Elements					
ferric chloride hexahydrate (FeCl_3_⋅6H_2_O)		0.05 mol (13.6 g)	—	0.05 mol (13.6 g)	0.05 mol (13.6 g)
aluminum nitrate nonahydrate (Al(NO_3_)_3_⋅9H_2_O)		—	0.05 mol (18.8 g)	0.05 mol (18.8 g)	0.05 mol (18.8 g)
zirconium chloride (ZrCl_4_)		—	—	—	0.05 mol (6.7 g)

“—“, the corresponding reagent was not added.

**Table 2:** Comprehensive set of experimental conditions.

	Conditions	pH	Dosage (mL/L)	Zr/Fe Molar Ratio	Sedimentation Time (h)
Experiments					
Effect of Zr/Fe molar ratio		5	25	0.2/1, 0.4/1, 0.6/1, 0.8/1, 1/1	2
Effect of dosage		5	10, 15, 20, 25, 30, 35, 40	0.6/1	2
Effect of pH		2, 3, 4, 5, 6, 7, 8, 9, 10, 11	25	0.6/1	2
Effect of sedimentation time		5	25	0.6/1	0.5, 1, 2, 4, 8, 12

**Table 3:** Characteristics of real rubber wastewater.

COD (mg/L)	TSS (mg/L)	Turbidity (NTU)	TP C_0_ (mg/L)	TP C_e_ (mg/L)	pH	Zeta Potential (mv)
61	224	5.5	110	0.44	7.4	−17.44

## Data Availability

The original contributions presented in this study are included in the article/Supplementary Material. Further inquiries can be directed to the corresponding authors.

## Supplementary Materials

The following supporting information can be downloaded at: https://www.mdpi.com/article/10.3390/ma19102049/s1. Section S1: Preparation of chemicals and reagents; Section S2: The distribution of the PSFAZ elements before and after coagulation; Figure S1: (1) EDS-elemental mapping of PSFCL before coagulation; EDS surface scan of (a) aluminum (b) iron; (c) oxygen; (d) silicon; (e) zirconium; (2) EDS-elemental mapping of PSFCL after coagulation; EDS surface scan of (a) aluminum (b) iron; (c) oxygen; (d) phosphorus; (e) silicon; Figure S2: Photograph of floc formation after treatment with PSFAZ. (Zr/Fe molar ratio = 0.03/0.05, pH = 5, dosage = 25 ml/L, sedimentation time = 2 h); Figure S3: Effect of sedimentation time towards phosphorus removal (Zr/Fe molar ratio = 0.6/1, pH = 6, dosage = 25 mL/L, sample size *n* = 3); Table S1: Coagulation capacities of phosphorus on metal-based flocculants and other state-of-the-art materials; Table S2: Cost analysis with other commercial coagulants. Refs. [60,61,62,63] can be found in Supplementary Materials.
