# Supplementary material for: Preparation of a Novel Zirconium-Loaded Flocculant for Efficient Removal of Phosphorus
Source: Materials (Basel). 2026 May 14;19(10):2049. doi: 10.3390/ma19102049 (PMC13208629; doi:10.3390/ma19102049)
Supplement: Supplementary file 1 [file materials-19-02049-s001.zip › materials-4269330-supplementary.pdf]

Supplementary Materials

Preparation of a Novel Zirconium-Loaded Flocculant for Efficient Removal of Phosphorus

Xueqing Xi [1], Xiang Li [1], Su fang He [1,2,3], Jiacheng Li [2,3,4], Boxuan Li [2,3,4*] and Xiangqian Zheng*[2,3,4,5]

[1] Faculty of Materials Science and Engineering, Kunming University of Science and Technology, Kunming 650093, Yunnan, China

[2] The Innovation Team for Volatile Organic Compounds Pollutants Control and Resource Utilization of Yunnan Province, Kunming 650500, P. R. China.

[3] The Higher Educational Key Laboratory for Odorous Volatile Organic Compounds Pollutants Control of Yunnan Province, Kunming 650500, P. R. China.

[4] Institute for inspection and certification of Xishuangbanna Dai Autonomous Prefecture, Jinghong 666100, P. R. China.

[5] Faculty of Chemical Engineering, Kunming University of Science and Technology, Kunming 650500, P. R. China.

* Corresponding author

E-mail: boxuanli9705@163.com (B. L.); 17373231245@163.com (X. Z.)

Section S1

Sodium silicate ($Na_2SiO_3 \cdot 9H_2O$) was obtained from Zhiyuan Chemical Reagent Co., Ltd. (Tianjin, China). Sodium hydroxide was purchased from Shanghai Aladdin Chemistry Co., Ltd. (Shanghai, China). Zirconium chloride ($ZrCl_4$), ferric chloride hexahydrate ($FeCl_3 \cdot 6H_2O$) and aluminum nitrate nonahydrate ($Al(NO_3)_3 \cdot 9H_2O$) were purchased from Keyuan Pharmaceutical Co. Ltd. (Jinan, China). Hydrochloric acid (36%) was provided by Kelong Chemical Co. Ltd. (Chengdu, China). Sodium dihydrogen phosphate (NaH_2PO_4) was purchased from Fuchen Chemical Co. Ltd. (Tianjin, China). Ultrapure water (R=18.3 MΩ/cm) was used for all the experiments. Simulated phosphorus wastewater was obtained by dissolving sodium dihydrogen phosphate to a certain amount. The initial concentration of phosphorus in the simulated wastewater was set as 400 mg/L. All reagents were of analytical grade and used without further purification.

Section S2

(1)

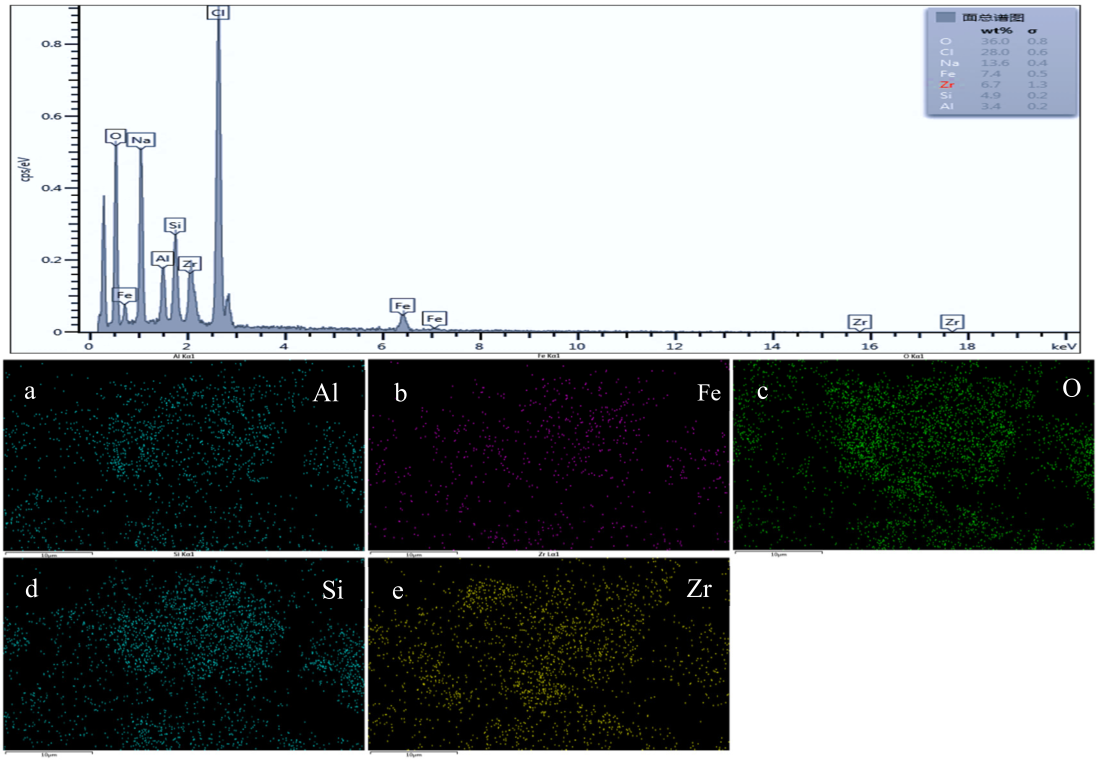

(2)

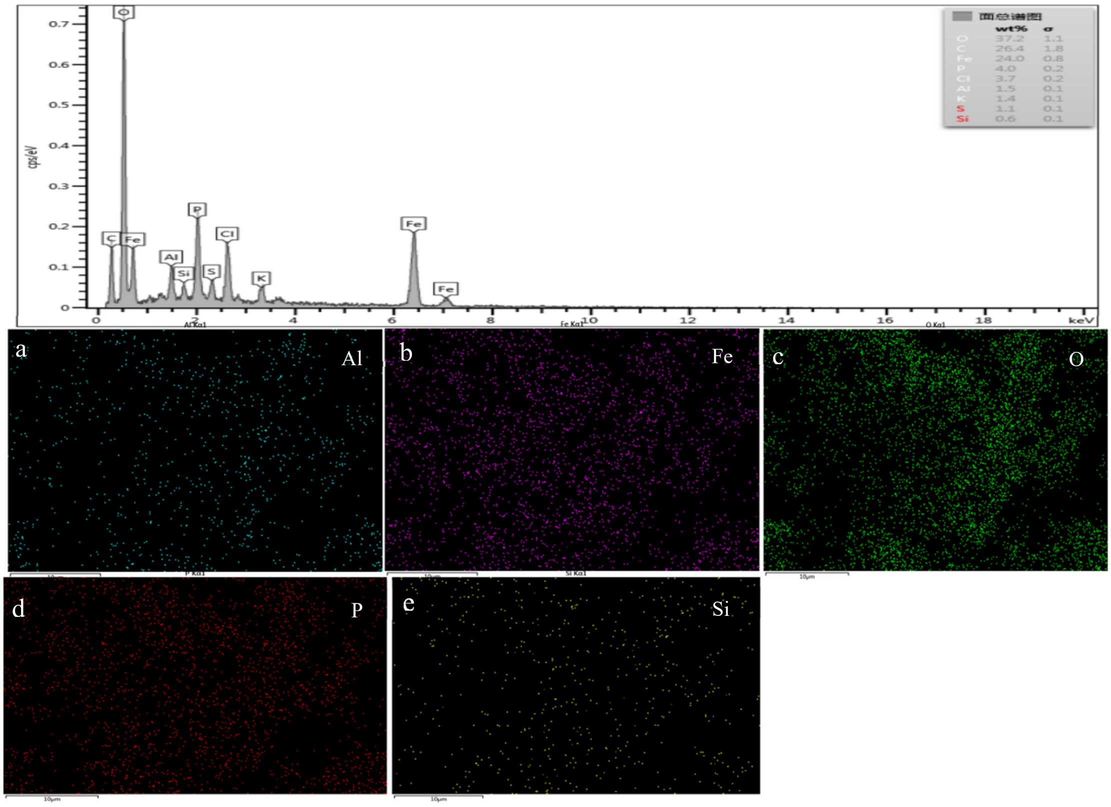

Figure S1. (1) EDS-elemental mapping of PSFCL before coagulation; EDS surface scan of (a) aluminum (b) iron; (c) oxygen; (d) silicon; (e) zirconium; (2) EDS-elemental mapping of PSFCL after coagulation; EDS surface scan of (a) aluminum (b) iron; (c) oxygen; (d) phosphorus; (e) silicon.

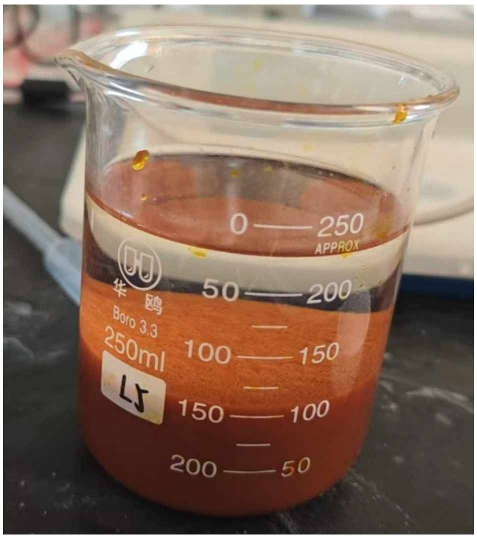

Figure S2. Photograph of floc formation after treatment with PSFAZ. (Zr/Fe molar ratio = 0.03/0.05, pH = 5, dosage = 25 ml/L, sedimentation time = 2 h).

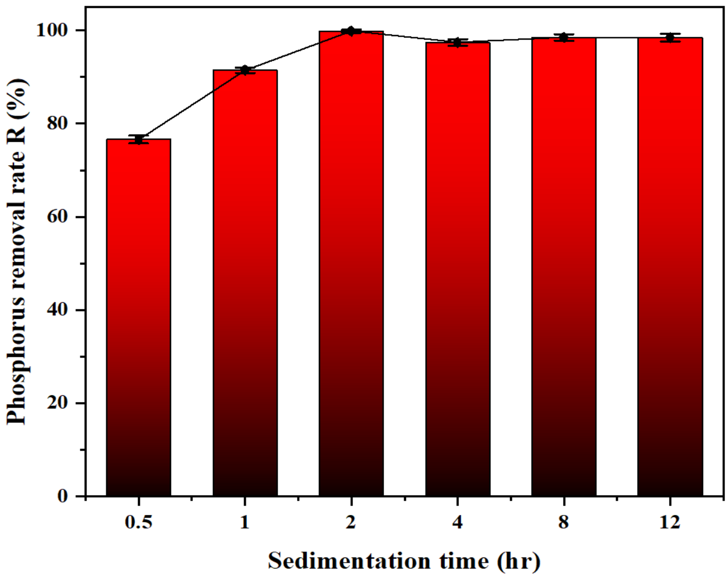

Figure S3. Effect of sedimentation time towards phosphorus removal (Zr/Fe molar ratio = 0.6/1, pH = 6, dosage = 25 mL/L, sample size *n* = 3).

Table S1. Coagulation capacities of phosphorus on metal-based flocculants and other state-of-the-art materials.

Samples	pH	Dose (mg/L)	TP C_0 (mg/L)	Rapid Stirring	Slow stirring	Settling time (min)	TP removal rate (%)	Ref.
PFASiC	5-9	50	10-50	150 rpm,3 min	30 rpm, 10min	30	> 95	[60]
PAC	6-8	50	10-50	150 rpm,3 min	30 rpm, 10min	30	94	[60]
FD-PSAF	2	10	31.3	120 rpm,2 min	60 rpm, 7 min	20	99.7	[61]
PFS	2	13	31.3	120 rpm,2 min	60 rpm, 7 min	20	98	[61]
CP/ATP-Fe(II)	3-9	250	75	/	/	60	90.1	[62]
RCS	4	625	125	/	/	120	≈100	[62]
RM-PCa	4	625	125	/	/	120	97.8	[63]
RM-ECa	4	625	125	/	/	120	98.1	[63]
PSFAZ	5	218	400	200 rpm, 2min	60 rpm, 5 min	120	99.3	The present study

PFASiC, polyaluminum ferric silicate chloride; PAC, polyaluminum chloride; FD-PSAF, polysilicate aluminum ferric from foundary dust; PFS, polymerized ferrous sulfate; CP/ATP-Fe(II), calcium peroxide/attapulgite-Fe(II); RCS, calcium carbide slag; RM-PCa, red mud-chemical calcium impregnation; RM-ECa, red mud-electrochemical precipitation loading calcium; PSFAZ, Polysilicate ferric aluminum zirconium; 218, the conversion factor of PSFAZ dose is 1 mL/L = 8.7 mg/L (in terms of metal species); TP, total phosphorus.

Table S2. Cost analysis with other commercial coagulants

Coagulant	Unit market price ($/ton)	Unit treatment cost (raw rubber wastewater, $/ton)
PAC	90-97	0.04-0.08
PFS	80-83	0.05-0.06

$FeCl_3$	160-167	0.05-0.09
PAFC	100-111	0.06-0.09
PAM	1667-1700	0.05-0.16
PSAF	100-125	0.09-0.11
PSFAZ	143	0.07

PAC, polyaluminum chloride; PFS, Polyferric sulfate; PAFC, Polyaluminum ferric chloride; PAM, Polyacrylamide; PSAF, Polymeric silicate aluminum ferric; PSFAZ, Polysilicate ferric aluminum zirconium. Source: Alibaba. Available online: https://www.alibaba.com (accessed on 2026-04-28).
